# Use of an Endobronchial Blocker in a Patient with Tracheobronchial Anomaly for Minimally Invasive Cardiac Surgery: A Case Report

**DOI:** 10.4274/TJAR.2024.231493

**Published:** 2024-02-28

**Authors:** Emine Nilgün Zengin, Nevriye Salman, Ayşegül Özgök

**Affiliations:** 1 Ankara Bilkent City Hospital, Clinic of Anaesthesiology and Reanimation, Ankara, Turkey

**Keywords:** Endobronchial blocker, minimally invasive cardiac surgery, one lung ventilation, tracheal bronchus, tracheobronchial anomaly

## Abstract

Tracheal bronchi (TB) is a rare anomaly and is usually asymptomatic. Although it is generally not a problem when a single lumen tube is used, it may cause ventilation difficulties in the intraoperative period in procedures requiring one lung ventilation, such as minimally invasive cardiac surgery. Therefore, these difficulties may cause intraoperative and postoperative complications. While a double-lumen tube is recommended as the primary choice for one-lung ventilation in patients with TB, bronchial blockers can be used to avoid the need for tube exchange in patients who will remain intubated in the postoperative period.

**Main Points**

• The frequency of minimally invasive cardiac surgery has increased in recent years and requires one lung ventilation.

• Tracheal bronchi are often incidentally diagnosed, posing challenges to endotracheal intubation and lung isolation, particularly during procedures requiring lung isolation.

• By diagnosing the tracheal bronchus in the pre-operative period, safe one-lung ventilation can be provided and possible postoperative pulmonary complications can be prevented.

## Introduction

Minimally invasive cardiac surgery (MICS) has become increasingly prevalent due to its advantages such as high patient satisfaction, favorable cosmetic outcomes, reduced postoperative pain, diminished stress response, decreased transfusion requirements, and accelerated recovery with a quicker return to normal activities.^1,2^ However, there are also disadvantages to this approach. Even in MICS patients with normal respiratory functions, postoperative atelectasis, pulmonary edema, and ventilation-perfusion mismatch can occur because of one-lung ventilation.^3^

The tracheal bronchus (TB) is a congenital anomaly defined as an abnormal bronchus originating from anywhere between the cricoid cartilage and the carina and directed toward the upper lobe of the lung. Although it can arise anywhere between the cricoid cartilage and the carina, it typically appears approximately 2 cm proximal to the carina.^4^ The prevalence of TB, usually on the right side, is a topic of debate. Various studies employing bronchoscopic, bronchographic, and computed tomography (CT) examinations have reported the presence of TB in approximately 0.1-1.3% of adults and 1.5-2% of the pediatric population. Although it rarely leads to recurrent infections in adults, it is often incidentally diagnosed on CT scans. In children, the presence of TB may manifest with symptoms such as recurrent pneumonia, stridor, or respiratory distress.^5^

Several classifications exist for the tracheal bronchi, but the most relevant for anesthesiologists is Conacher’s classification based on the anatomical relationship between the TB and the carina.^6^ Conacher identified three types based on this relationship:^7^ Type I, where the TB is ≥2 cm from the carina and the distal trachea is narrowed; Type II, where the TB is ≥2 cm from the carina and the distal trachea has a normal diameter; and Type III, where the TB is at or near the level of the carina.

Although tracheal bronchi are often clinically asymptomatic, they may present symptoms when OLV is required. Complications such as atelectasis, hypoxemia, and barotrauma can arise when TB is occluded.^6,8^ In some cases, it can lead to inadequate lung isolation.^6,9^

This case report aims to share the use of endobronchial blockers (EBB) in a patient with tracheal bronchial anomaly, highlighting the importance of addressing such anomalies in MICS.

## Case Presentation

**Figure 1:**
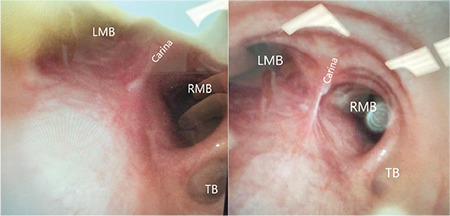
Fiberoptic bronchoscopy image of the patient. The tracheal bronchus was determined as type III according to Conacher’s classification. LMB, left main bronchus; RMB, right main bronchus; TB, tracheal bronchus.

A patient undergoing minimally invasive coronary artery bypass grafting (CABG) through informed and consented left thoracotomy was evaluated for perioperative findings. The patient, a 53-year-old man with an ASA physical status of II, weighing 73 kg and measuring 172 cm, had a history of smoking and diabetes as the only comorbidity. Standard ASA monitoring was initiated. The following induction, endotracheal intubation was performed using a size 9 tube. When attempting to place a 7 F EBB using fiberoptic bronchoscopy (FOB), TB was observed to the right of the right main bronchus (Figure 1). EBB (Hangzhou Tappa Medical Technology CO., Hangzhou, China) was successfully placed in the left main bronchus without difficulty. The cross-clamp time was 114 min, the cardiopulmonary bypass time was 151 min, and the anesthesia duration was 450 min. A CABGx3 procedure was performed. The EBB was removed at the end of surgery. The patient was transferred to the intensive care unit (ICU). The patient was monitored in the ICU for 1 day, followed by 5 days in the general ward, and was discharged without complications.

## Discussion

**Figure 2:**
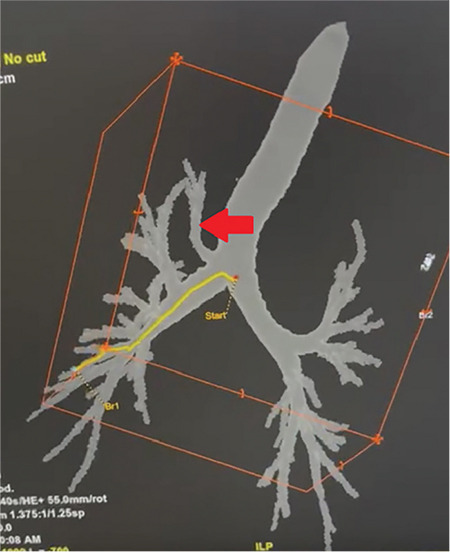
Computed tomography image of our patient's tracheal bronchus. The tracheal bronchus (red arrow) is located as the right apical posterior segment.

Tracheal bronchi often receive incidental diagnoses, as in this case, and can complicate lung isolation, rendering it inadequate.^10^ During endotracheal intubation, bronchial lumen occlusion can lead to atelectasis, pneumonia, respiratory failure, and inadequate ventilation.^11^ To address this, particularly in right lung isolation, tools such as the Fogarty embolism catheter, double-lumen tube (DLT), or Rüsch EZ-Blocker can be used.^6,10^ Our patient’s preoperative CT scan revealed no abnormalities, except for linear atelectasis in the left middle lobe (Figure 2). Therefore, the diagnosis of TB was established intraoperatively with bronchoscopic guidance during BB placement. Despite this, we did not encounter any issues with placing the left EBB or maintaining anesthesia. The patient was extubate at the sixth postoperative hour, and discharge occurred without the development of pulmonary complications.

Postoperative pulmonary complications are commonly cited as significant causes of morbidity and mortality, particularly after major surgeries.^12^ Most MICS procedures performed via thoracotomy involve OLV, requiring the patient to be positioned supine or with a slightly elevated hemithorax, both of which increase the risk of hypoxemia. Therefore, a thorough respiratory system assessment is necessary.^2,13^ Although our patient did not have chronic lung disease, the presence of TB suggests an increased risk of respiratory complications. Nevertheless, lung isolation using an EBB under FOB guidance, along with the implementation of a lung-protective ventilation strategy, mitigated postoperative respiratory distress.

Rarely encountered, TB, especially when unnoticed in situations requiring specialized airway management, can negatively impact patients’ recovery processes.^11^ These situations include the methods for lung isolation necessary for OLV. In patients with TB, the primary choice for ensuring successful OLV is the use of a DLT.^14^ While a well-placed left DLT may not pose issues in patients with TB, problems may arise if the TB’s anatomical localization does not align with the tracheal opening of the DLT, leading to difficulty in ventilating the right upper lobe. Although this may not present problems in the early stages, shunt-related hypoxemia and postoperative pulmonary complications may arise in the later stages of surgery. However, the use of BB is advantageous over DLT in difficult intubation situations in small adults or children and eliminates the need for a tube exchange when postoperative mechanical ventilation is essential.^14^ In our clinic, where we have experience with both FOB and EBB in MICS, we prefer the use of EBB. This preference is based on the fact that EBB eliminates the need to change endotracheal tubes for postoperative ventilation, resulting in less airway trauma.^15^

This issue can also be problematic not only in cases requiring lung isolation but also in surgeries performed using a single-lumen tube. If TB is located more proximal in the trachea and a tracheal tube is advanced more distally, patients may not present with intraoperative symptoms but may increase the risk of right upper lobe atelectasis and pneumonia in the postoperative period.

## Conclusion

In MICS, while a comprehensive respiratory system assessment is performed, it is crucial to conduct detailed CT evaluations. Even if anomalies, such as TB, are not detected radiologically in patients undergoing OLV, they can be identified through FOB application, thereby preventing potential complications.
